# Filling the Gap: Simulation-based Crisis Resource Management Training for Emergency Medicine Residents

**DOI:** 10.5811/westjem.2017.10.35284

**Published:** 2017-12-14

**Authors:** Jessica R. Parsons, Amanda Crichlow, Srikala Ponnuru, Patricia A. Shewokis, Varsha Goswami, Sharon Griswold

**Affiliations:** * Drexel University College of Medicine, Department of Emergency Medicine, Philadelphia, Pennsylvania; † University of Florida College of Medicine, Department of Emergency Medicine, Jacksonville, Florida; ‡ Drexel University College of Nursing and Health Professions, Department of Nutrition Sciences & School of Biomedical Engineering, Science and Health Systems, Philadelphia, Pennsylvania

## Abstract

**Introduction:**

In today’s team-oriented healthcare environment, high-quality patient care requires physicians to possess not only medical knowledge and technical skills but also crisis resource management (CRM) skills. In emergency medicine (EM), the high acuity and dynamic environment makes CRM skills of physicians particularly critical to healthcare team success. The Accreditation Council of Graduate Medicine Education Core Competencies that guide residency program curriculums include CRM skills; however, EM residency programs are not given specific instructions as to how to teach these skills to their trainees. This article describes a simulation-based CRM course designed specifically for novice EM residents.

**Methods:**

The CRM course includes an introductory didactic presentation followed by a series of simulation scenarios and structured debriefs. The course is designed to use observational learning within simulation education to decrease the time and resources required for implementation. To assess the effectiveness in improving team CRM skills, two independent raters use a validated CRM global rating scale to measure the CRM skills displayed by teams of EM interns in a pretest and posttest during the course.

**Results:**

The CRM course improved leadership, problem solving, communication, situational awareness, teamwork, resource utilization and overall CRM skills displayed by teams of EM interns. While the improvement from pretest to posttest did not reach statistical significance for this pilot study, the large effect sizes suggest that statistical significance may be achieved with a larger sample size.

**Conclusion:**

This course can feasibly be incorporated into existing EM residency curriculums to provide EM trainees with basic CRM skills required of successful emergency physicians. We believe integrating CRM training early into existing EM education encourages continued deliberate practice, discussion, and improvement of essential CRM skills.

## BACKGROUND

High-quality medical care of patients in the emergency department (ED) is dependent on teams of qualified healthcare providers. Emergency physicians have critical roles in the functioning of these teams. They must possess the medical knowledge and clinical skills needed to diagnose and treat high acuity medical conditions, but they must also possess the teamwork and interpersonal skills needed to successfully coordinate efficient team-based care in high-stakes situations. The skills that contribute to leadership, teamwork, communication, resource utilization and problem solving are frequently referred to as crisis resource management (CRM) skills and are increasingly recognized as factors that impact patient safety in acute healthcare fields such as emergency medicine (EM). 1–5 Conversely, the lack of CRM skills has been implicated in adverse patient outcomes and malpractice cases. 5

In the current era of outcomes-based medical education, the American Council for Graduate Medical Education (ACGME) requires EM residency programs to ensure trainees meet core competencies. The core competencies entitled “Interpersonal and Communication Skills” and “Systems-based Practice” involve various CRM skills such as teamwork, communication and resource utilization. 6 While residency programs are expected to teach and evaluate these skills, the ACGME does not specify how this training should be incorporated into residency education, and as a result many curriculums lack a dedicated plan for teaching these less tangible skills.

Historically, residents have been expected to learn CRM skills through role modeling, mentorship or trial and error using an apprentice-style approach to medical training. 7 However, better understanding of CRM suggests that these skills are teachable and measurable through more explicit approaches, similar to the medical knowledge and procedural skills taught across all residency programs. 8 To effectively equip trainees with the complete skillset needed to be successful, dedicated curricula designed to introduce, teach and reinforce CRM skills are warranted in residency education.

Several standardized team-training programs, such as “MedTeams” and “TeamStepps,” have been developed and disseminated nationally.^5^,^9^ The broad CRM skills in these courses are applicable to a wide variety of healthcare settings and disciplines. However, specific healthcare environments such as the ED and specific learners such as EM residents may warrant specialized CRM skills training.^10^,^11^

## OBJECTIVES

Our first objective was to design a CRM course encompassing basic CRM principles critical to the practice of EM that could be feasibly incorporated into any EM residency curriculum. Our second objective was to evaluate the CRM course’s efficacy in helping novice EM residents develop CRM skills. We conducted a pilot study to measure improvement in CRM skills displayed by teams of EM interns during the course.

## CURRICULAR DESIGN

We conducted a literature review to explore existing courses that encompass CRM principles and determine which CRM skills should be included in this course. Despite discovering extensive lists of CRM skills pertinent to EM, 10–12 our search revealed just one previous CRM curriculum specifically designed for EM residents. 13 While the course was well received by participants, the authors did not attempt to measure the course’s effectiveness in improving CRM skills.

Our CRM course consists of two key components: 1) an introductory lecture presentation, and 2) a series of six specialized simulation scenarios. The 30-minute lecture introduces the key concepts and history of CRM in aviation and in healthcare and highlights the increasing recognition of CRM’s role in patient safety. The presentation also describes the specific roles of the leader and ED team members, and defines basic CRM terms such as “closed loop communication,” “shared mental model,” “workload management,” and “situational awareness.” Establishing this baseline knowledge aids in the discussions of CRM during debriefs of simulation scenarios and in the ED.

The majority of the CRM training involves a series of six high-fidelity simulation scenarios followed by structured debriefs. Various studies suggest that simulation is an ideal educational modality to teach and evaluate CRM skills.^7^,^10^,^14^,^15^ Simulation not only introduces the importance of CRM skills, but also allows residents to deliberately practice the CRM skills in a safe environment where no patients are at risk.

The simulation cases in this course provide high-acuity EM situations that require effective CRM skills. The cases are designed to mimic the intense time pressures, rapidly evolving situations and high-acuity illnesses that are routinely experienced in the ED.^10^,^16^ We chose all cases from our department’s existing simulation case bank and modified them to meet specific learning objectives. The six cases used during this pilot course are described in the Supplemental Table. While each case has specific objectives for the medical management of each diagnosis, all cases incorporate the uniform CRM objectives listed in Table 1.

Each simulation scenario runs for 10–15 minutes followed by a 30-minute debrief by a single facilitator. The scenarios use either a standardized patient actor or the SimMan3G patient simulator, and cast simulation faculty as the nurse, family member or consultant. Following each scenario, a debrief is conducted by a facilitator and addresses both the medical management learning objectives, and the uniform CRM learning objectives within each scenario.

A randomized control study conducted in anesthesia CRM training showed that observers of a simulation scenario can gain the same improvement in CRM skills as active participants in the scenario. 17 With this in mind, our CRM course includes active participation in some scenarios as well as observation of other scenarios. More specifically, all teams participate separately in Case 1 and Case 6. Team performances in Case 1 provide pretest data, and team performances in Case 6 provide posttest data to measure the impact of the overall course. For Cases 2, 3, 4, and 5, only one team participates in the scenario while the remaining teams are observers. Observing teams are instructed to take notes on both the medical management and the CRM of the case. The facilitator then engages both the participating team and the observing teams in discussion during the debrief. The use of observational learning in four of the six cases minimizes the need for multiple simulation rooms, larger simulation and debriefing staff and time that would be required to allow every team to participate in every case separately.

Finally, to recreate the ad hoc nature of teams in the ED setting, this course randomly distributes residents into small teams at three separate times over the two-week period. Random and frequent shuffling of the teams recreates a more realistic working environment for our trainees and allows them to work with different peers in the various simulation cases.

## IMPACT/EFFECTIVENESS

The pilot study of this course involved a cohort of 14 EM interns who participated in the simulation scenarios during four separate days in July 2016. This CRM course was a component of a larger two-week curriculum designed to provide an introduction to residency and to the clinical practice of EM. The institutional review board of our institution reviewed this study and determined that it was exempt from requiring informed consent of participants or ongoing review. The interns were randomly divided into four teams and these teams were redistributed three times over the course to replicate the ad hoc nature of ED teams. By the end of the course, each intern participated in a total of three cases and observed three additional cases.

During every simulation scenario, the CRM skills demonstrated by the participating team were evaluated by two independent raters using the Ottawa CRM Global Rating Scale (GRS). This CRM evaluation tool has been shown to have acceptable construct validity and interrater reliability^18^,^19^ and has previously been used to measure the development of CRM skills in EM residents. 15 The Ottawa GRS provides a seven-point scale to evaluate the overall CRM performance and CRM skills in five specific categories: leadership, problem solving, situational awareness, resource utilization, and communication. Two trained EM faculty, both with extensive expertise in simulation education, acted as independent raters throughout the course. Both raters had used the Ottawa GRS in one prior study but did not receive any extensive training in the use of the tool. The same two raters were present for all simulation scenarios during the course.

Results of this pilot study suggest that this CRM course was effective in improving CRM skills among teams of novice interns; however, the improvements were not statistically significant. The median pretest and posttest scores and the data analysis for overall CRM scores and CRM category scores are presented in Table 2. We used Number Cruncher Statistical Software for the analyses. 20 Given that the unit of analysis is teams of interns and our sample size was N=4, we established a significance criterion of α= 0.05. To control for Type I errors, we conducted Bonferroni corrections (corrected p-value = number of comparisons/α = 6/0.05 = 0.0083).^21^

A Wilcoxon-signed rank test showed that the posttest scores for overall CRM performance were not statistically significantly higher than the pretest scores when using the Bonferroni corrected p-values. (All p-values were greater than the corrected p = 0.0083.) Similar data analysis for each category of CRM skills measured by the Ottawa GRS also demonstrated an improvement from pretest to posttest but also failed to meet statistical significance.

We calculated effect sizes to further aid in the interpretation of the data. The |r| effect size index was interpreted similar to a correlation coefficient |r|, with |r| = 0.10, 0.30 and > 0.50 interpreted as small, medium and large effects, respectively.^22^ The improvements in overall CRM and each CRM category all showed large effect sizes ranging from |r| = 0.581 to 0.601. The large effect sizes detected for each dependent measure implies that the posttest scores would reflect a significant improvement over the pretest scores with an increased sample size.

We conducted an interrater reliability analysis using a kappa statistic with linear weighting to determine consistency between the two faculty raters. A single analysis of all pretest and posttest scores by the raters was used in the calculation of the kappa statistic. The interrater reliability for the raters was found to be kappa = 0.74, 95% CI (0.66, 0.82). This kappa value represents good agreement between the two raters in this study. 23

While pretest and posttest data were analyzed to determine the effectiveness of the CRM training, we also evaluated the CRM skills during Cases 2, 3, 4, and 5 to explore the potential value of the observational learning in this course. These scores were not included in the data analysis since only a single team actively participated in each of these cases while the remaining three teams observed. As shown in the figure below, each team showed an overall gradual improvement in CRM skills compared to the preceding teams, suggesting that observational learning of CRM was effective in this setting.

The results support previous evidence that simulation is an effective educational modality to teach CRM skills and that observational learning in simulation is an effective tool to optimize training and minimize the time and resources required. Furthermore, this course can be easily incorporated into existing EM residency educational curriculums since it is designed to be feasible with one simulation room, a single simulation debriefing facilitator, and minimal other staff or faculty to serve as confederates in the scenarios. It is an option for any EM residency without access to a large simulation center.

While these results suggest that CRM skills can be significantly improved through a short simulation-based educational intervention, there are a number of limitations. This study is limited by the small sample size and the setting of a single EM residency program. The CRM principles introduced and evaluated in this course reflect just a subset of those CRM skills needed for EM. It is our belief that adding basic CRM to the vocabulary and skillset of an EM intern sets the stage for continued appreciation, deliberate practice, and ongoing improvement of CRM skills during training and throughout a career in EM.

This pilot study also does not assess the retention of the CRM skills beyond the two-week course. Additional testing over multiple years of residency training would be valuable in detecting improvement and retention of CRM skills over time. Ultimately, further studies would be beneficial to determine if the CRM skills achieved through simulated scenarios affect behaviors or success of residents in the ED.

## Supplementary Information



## Figures and Tables

**Figure:**
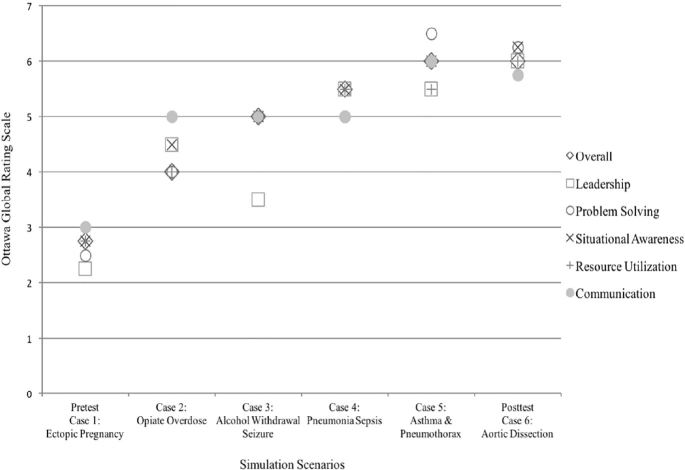
Crisis Reseorce Management team performance during simulation scenarios

**Table 1:** Simulation scenario crisis resource management objectives.

Category	Objective
Objective	Team member(s) verbally identify the leader within the first minute.
	The leader maintains a global perspective of the scenario.
Problem solving	Team member(s) verbalize a differential diagnosis prior to completion of the scenario.
Situational awareness	Team member(s) verbalize abnormal vital signs or significant changes in vital signs within two minutes
	A summary of the situation and plan going forward is verbalized for the entire team to hear (shared mental model).
Resource utilization	Tasks are clearly divided between members of the team.
	Consultant is provided with appropriate summary and specific requests for actions.
Communication	Team member(s) consistently use closed loop communication.
	Input from team members is elicited and considered.

**Table 2:** Pretest and posttest median scores using the Ottawa Crisis Resource Management Global Rating Scale (N=4) including descriptive and inferential statistics and effect sizes.

Dependent measure	Case 1-pretest (Mdn + IQR)	Case 6-posttest (Mdn + IQR)	Z-value	p-value*	|r|
Overall score	2.75 + 1.25	6.00 + 0.38	−1.657	0.049	0.586
Leadership	2.25 + 1.25	6.00 + 0.75	−1.657	0.049	0.586
Problem solving	2.50 + 2.25	6.25 + 0.88	−1.643	0.050	0.581
Situational awareness	2.75 + 0.88	6.25 + 1.63	−1.643	0.050	0.581
Resource utilization	2.75 + 1.63	6.00 + 0.75	−1.701	0.044	0.601
Communication	3.00 + 0.75	5.75 + 0.50	−1.657	0.049	0.586

*Mdn*, Median; *IQR*, Interquartile Range; |*r*|, effect size.

* All p-values are not significant with Bonferroni correction for Type I error rate inflation.
