# Supplementary material for: Filling the Gap: Simulation-based Crisis Resource Management Training for Emergency Medicine Residents
Source: West J Emerg Med. 2017 Dec 14;19(1):205–10. doi: 10.5811/westjem.2017.10.35284 (PMC5785195; doi:10.5811/westjem.2017.10.35284)
Supplement: Supplementary file 1 [file wjem-19-205-s001.docx]

Supplemental Table. Summary of simulation scenarios used during the Crisis Resource Management course

|  | Diagnosis:  Summary of Patient Presentation | Medical Management Critical Actions |
| --- | --- | --- |
| Case 1 Pretest | Ruptured Ectopic Pregnancy:  28 year old female presenting with right lower quadrant abdominal pain and syncope. She has hypotension, tachycardia, and severe tenderness with palpation of right lower quadrant. | - Initiate IVF resuscitation within two minutes. - Obtain a urine or serum pregnancy test. - Perform a bedside FAST exam. - Consult obstetrics for emergency laparotomy. - Crossmatch blood for transfusion. |
| Case 2 | Opiate Overdose:  34 year old male brought by EMS after being found unresponsive on the sidewalk. He is bradycardic, hypoxic, and bradypneic. He has miosis. | - Initiate oxygen administration within two minutes. - Perform an accucheck within two minutes. - Obtain IV access (nurse is unable to obtain peripheral IV access on patient’s arms). - Administer small dose(s) of naloxone to correct hypoxia. |
| Case 3 | Alcohol Withdrawal Seizure:  48 year old male brought by EMS after seizure witnessed by bystanders in the street. He is hypertensive, tachycardic, and tremulous but alert and oriented. Four minutes into scenario, he has a generalized tonic clonic seizure lasting one minute. | - Perform an accucheck within two minutes. - Obtain history of alcohol abuse and withdrawal seizures from patient. - Administer appropriate benzodiazepines. - Admit patient to the ICU. |
| Case 4 | Pneumonia Sepsis:  78 year old female is sent from nursing home for lethargy. She is febrile, tachycardic, tachypneic, and mildly hypotensive. | - Administer supplemental oxygen within two minutes. - Order 30ml/kg isotonic IVF. - Order blood cultures and lactate. - Identify right middle lobe infiltrate on chest x-ray. - Order broad spectrum antibiotics. |
| Case 5 | Asthma Exacerbation with Pneumothorax:  33 year old female with history of asthma presenting sudden onset of left-sided chest pain and dyspnea. Patient has diffuse wheezing and accessory muscle use. | - Initiate inhaled bronchodilators within two minutes. - Initiate non-invasive ventilation support within six minutes. - Verbalize risk stratification for pulmonary embolism. - Identify pneumothorax on chest x-ray. - Verbalize placement of thoracostomy tube or pigtail catheter. |
| Case 6 Posttest | Aortic Dissection:  64 year old male presenting with severe substernal chest pain that radiates to his back. He has severe hypertension and mild tachycardia. EKG shows STEMI. | - Activate a STEMI alert within two minutes. - Obtain a chest x-ray. - Verbalize widened mediastinum on chest x-ray. - Consult cardiothoracic surgery for concern for aortic dissection. - Initiate appropriate anti-hypertensive medications |

*IVF*, intravenous fluid; *FAST*, Focused Assessment with Sonography for Trauma; *EMS*, Emergency Medical Services; *EKG*, electrocardiogram; *ICU*, intensive care unit; *STEMI*, ST-elevation myocardial infarction
